# Dissecting the chromosome-level genome of the Asian Clam (*Corbicula fluminea*)

**DOI:** 10.1038/s41598-021-94545-2

**Published:** 2021-07-22

**Authors:** Tongqing Zhang, Jiawen Yin, Shengkai Tang, Daming Li, Xiankun Gu, Shengyu Zhang, Weiguo Suo, Xiaowei Liu, Yanshan Liu, Qicheng Jiang, Muzi Zhao, Yue Yin, Jianlin Pan

**Affiliations:** 1 Freshwater Fisheries Research Institute of Jiangsu Province, Nanjing, China; 2 Hongze Lake Fisheries Administration Committee Office of Jiangsu Province, Huai’an, China; 3 Fisheries Management Commission of Gehu Lake, Changzhou, China

**Keywords:** Bioinformatics, Genomic analysis, Sequencing

## Abstract

The Asian Clam (*Corbicula fluminea*) is a valuable commercial and medicinal bivalve, which is widely distributed in East and Southeast Asia. As a natural nutrient source, the clam is rich in protein, amino acids, and microelements. The genome of *C. fluminea* has not yet been characterized; therefore, genome-assisted breeding and improvements cannot yet be implemented. In this work, we present a de novo chromosome-scale genome assembly of *C. fluminea* using PacBio and Hi-C sequencing technologies. The assembled genome comprised 4728 contigs, with a contig N50 of 521.06 Kb, and 1,215 scaffolds with a scaffold N50 of 70.62 Mb. More than 1.51 Gb (99.17%) of genomic sequences were anchored to 18 chromosomes, of which 1.40 Gb (92.81%) of genomic sequences were ordered and oriented. The genome contains 38,841 coding genes, 32,591 (83.91%) of which were annotated in at least one functional database. Compared with related species, *C. fluminea* had 851 expanded gene families and 191 contracted gene families. The phylogenetic tree showed that *C. fluminea* diverged from *Ruditapes philippinarum*, ~ 228.89 million years ago (Mya), and the genomes of *C. fluminea* and *R. philippinarum* shared 244 syntenic blocks. Additionally, we identified 2 MITF members and 99 NLRP members in *C. fluminea* genome. The high-quality and chromosomal Asian Clam genome will be a valuable resource for a range of development and breeding studies of *C. fluminea* in future research.

## Introduction

The Asian Clam (*Corbicula fluminea*) belongs to the family Corbiculidae, genus *Corbicula*^1,2^.The Asian Clam has a round base and triangular double shells. The surface of the shells is glossy, and the shell color varies with the living environment^3^. Shells are brown, yellow, green, or black and are characterized by circular growth lines^4^. There are three main teeth in the left shell, one in the front, one in the back and one in the side^5^. The Asian Clam has undergone the planktonic larvae stage, grows rapidly and takes only 73–91 days for sexual maturation^6,7^. They are widely distributed in lakes and rivers in China, and play an important impact on the diversity of freshwater ecosystems^8^. The native distribution of *C. fluminea* is Asia, the Middle East, Africa and Australia^9^. In foreign countries, *C. fluminea* was first recorded as in the early twentieth century^10^. They may have spread worldwide by carried as food resource /unintentionally attaching to the hull or though ballast waters, then occupying rivers and lakes and becoming alien invasive species in American and European ecosystems^11–13^.

As a local delicacy, the meat of *C. fluminea* is nutritious. It is rich in protein, essential amino acids, taurine, active peptides, vitamins and microelements^14,15^. According to the Compendium of Materia Medica, the Asian Clam has medicinal applications of detumescence, dehumidification, sobering up, and benefits to the liver^16^. Modern research has found that the *Corbicula* extracts can protect against liver damage and reduce blood lipids^17^. Compared with Japan and South Korea, the deep processing ability for the Asian Clam in China is underdeveloped, resulting in its economic and medicinal value not being fully exploited^18^.

The Asian Clam as a benthic bivalve is critical in bioturbation, bioirrigation, and the breakdown of organic matter^19^. It displays strong environmental adaptability, reproductive capacity and diffusion ability^20^. The characteristic of the Asian Clam for the tolerance for diverse biotic and abiotic factors, such as antibiogram, heavy metal tolerance, hypoxia, have attracted great attention in the recent years^21^. The Asian Clam has a robust and multifaceted immune system, which is strong enough to cope with all kinds of harsh living environments^22^. The underlying molecular mechanisms of mollusks for immune response and reproductive capacity still undergo a slow development, resulting in these processes is still very limited in *C. fluminea.* Deciphering the genome of *C. fluminea* is the most basic step in our research program. The acquisition of a high-quality genome may provide more detailed insights into the value of *C. fluminea*. During the past decade, whole-genome sequencing has been widely performed on a number of Mollusca due to the rapid development of third-generation sequencing^23,24^. However, only 0.04% of the species described in Mollusca have available genome assemblies^25^. As the second most species-rich phylum^26^, the amount of Mollusca whole genomes is still low and the assembly of their genomes still needs to move forward. In present study, a de novo genome sequencing of *C. fluminea* was performed*,* and this genome may provide the foundation for a range of development and breeding studies of *C. fluminea* in future research.

## Results

### Genome sequencing assessment

A total of 252.77 Gb of clean data were generated with the Illumina HiSeq X Ten platform, and the data covered the depth of 154.13X for the Asian Clam genome (Table S1). Two single-molecule real-time (SMRT) cells were responsible for producing data from PacBio Sequel platform, and approximately 15.03 million PacBio reads (∼ 293.72 Gb, 193.40 X) were generated (Table S1). The max subread for PacBio was 286.39 kb; the N50 and mean length of subreads were 31.18 kb and 19.54 kb, respectively. Two libraries for the high-throughput chromosome conformation capture technology (Hi-C) were employed, yielding a total of 780.87 million clean reads (~ 233.26 Gb, 142.23X) (Table S1). Additionally, approximately 8 Gb clean data of transcriptomic data was obtained for genome annotation.

### Genome estimation and assembly

The k-mer analysis yielded more than 187.45 billion k-mers, which was used to calculate the genome size. The main peak of k-mer was the depth of 115, from which the genome size was estimated to be ~ 1.64 Gb (Fig. S1). The k-mer depths of 58 and 230 estimated a heterozygosity rate of 2.41% and a repeat ratio of 64.55% for the Asian Clam genome, respectively.

The 15.03 million subreads from PacBio platform entered the workflow of Canu for polishing. Canu and SMART denovo assembled the subreads individually and then merged the results. After contig assembly and error-corrected procedures, the initial 4,347contigs were obtained. The draft genome assembly of Asian Clam resulted in a genome size of 1.52 Gb, with a contig N50 size of 603.64 Kb.

### Chromosome construction by Hi-C

A total of 571.60 million read pairs (73.20%) of total Hi-C data were mapped to the draft genome assembly, and 116.65 million valid interaction pairs (14.94%) played a role in the assembly (Table S2). The contigs of the draft genome (4347contigs) were broken and reassembled using the valid interaction pairs, yielding 4728 corrected contigs. The final assembly presented a high-quality genome of the *C. fluminea* that reached 1.52 Gb in length, and it was characterized by a contig N50 of 521.06 Kb and a scaffold N50 of 70.62 Mb. The final genome comprised 1215 scaffolds, and the mix contig and scaffold were 3.17 Mb and 144.27 Mb, respectively.

**Figure 1:**
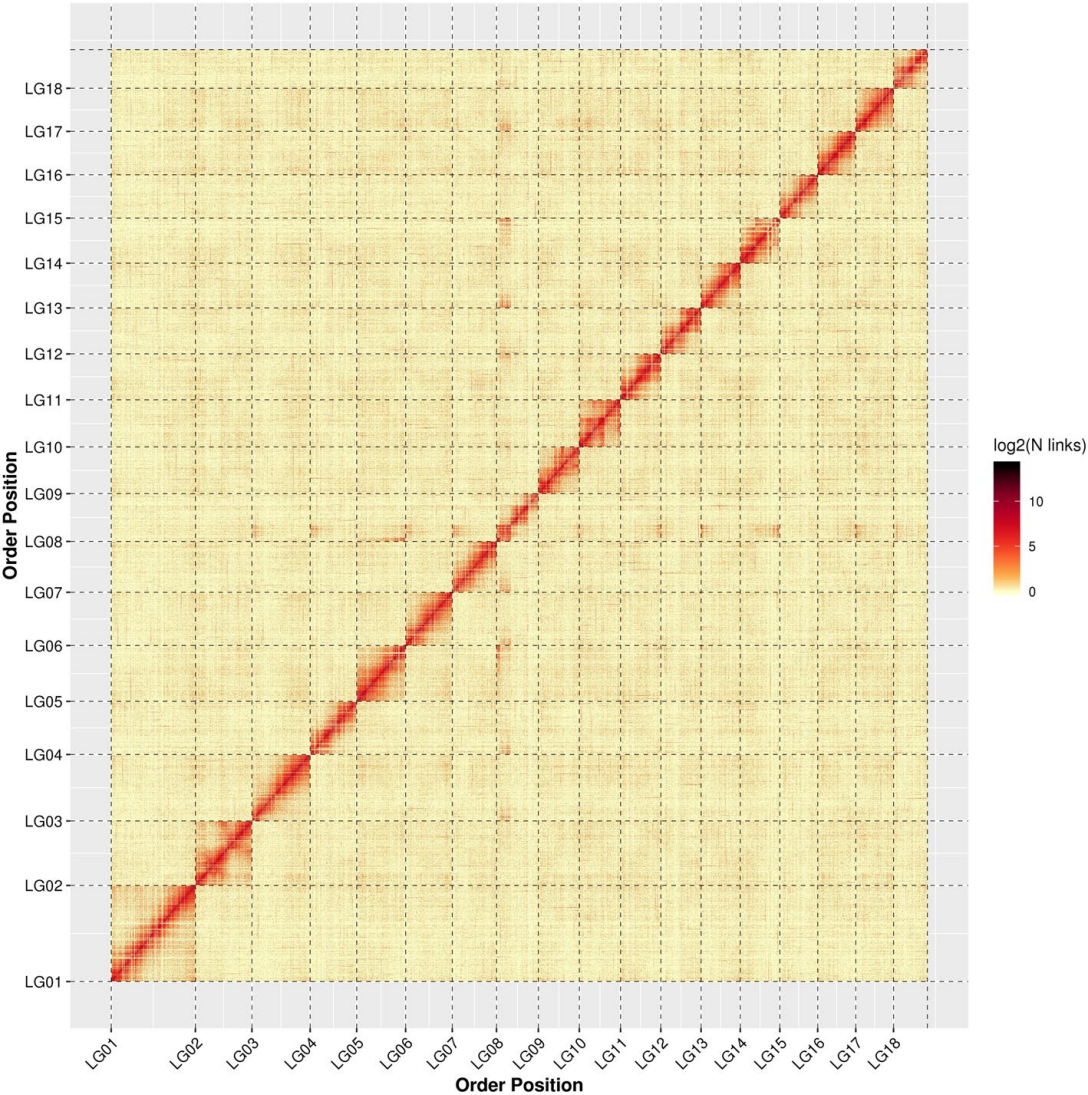
The genome-wide Hi-C heatmap of *Corbicula fluminea*. LG1-18 are the abbreviations of Lachesis Groups 1–18 representing the 18 pseudochromosomes.

The high-throughput chromosome conformation capture technology (Hi-C) dissected the classification, combination and order of contigs inside the genome of Asian Clam. A total of 1.51 Gb of genomic sequences accounting for 99.17% of total sequences, were assigned to 18 haploid chromosomes (Fig. 1). Among the 4728 corrected contigs, 4621 contigs (97.74%) were anchored onto 18 haploid chromosomes. Additionally, 1.40 Gb (92.81%) of genomic sequences were anchored with a defined order and orientation (Table S3).

### Evaluation and repetitive genome elements

The BUSCO data showed the Asian Clam genome covered 86.65% of the complete core genes (Table S4). The 97.45% of Illumina reads successfully mapped back to the assembly, indicating the high degree of completeness of the Asian Clam genome. More than 1.06 Gb of genomic sequences were identified and marked as repeats, representing 69.66% of the total genomic sequences. Approximately 608.85 Mb (57.54%) of the Asian Clam genome consisted of Large retrotransposons derivatives (LARDs), which was the predominant repeat. Terminal inverted repeats (TIRs), Penelope-like elements (PLEs), and Long interspersed nuclear elements (LINEs) comprised 10.46%, 12.38%, and 7.07% of the Asian Clam genome, respectively (Table S5).

### Gene prediction and gene annotation

**Figure 2:**
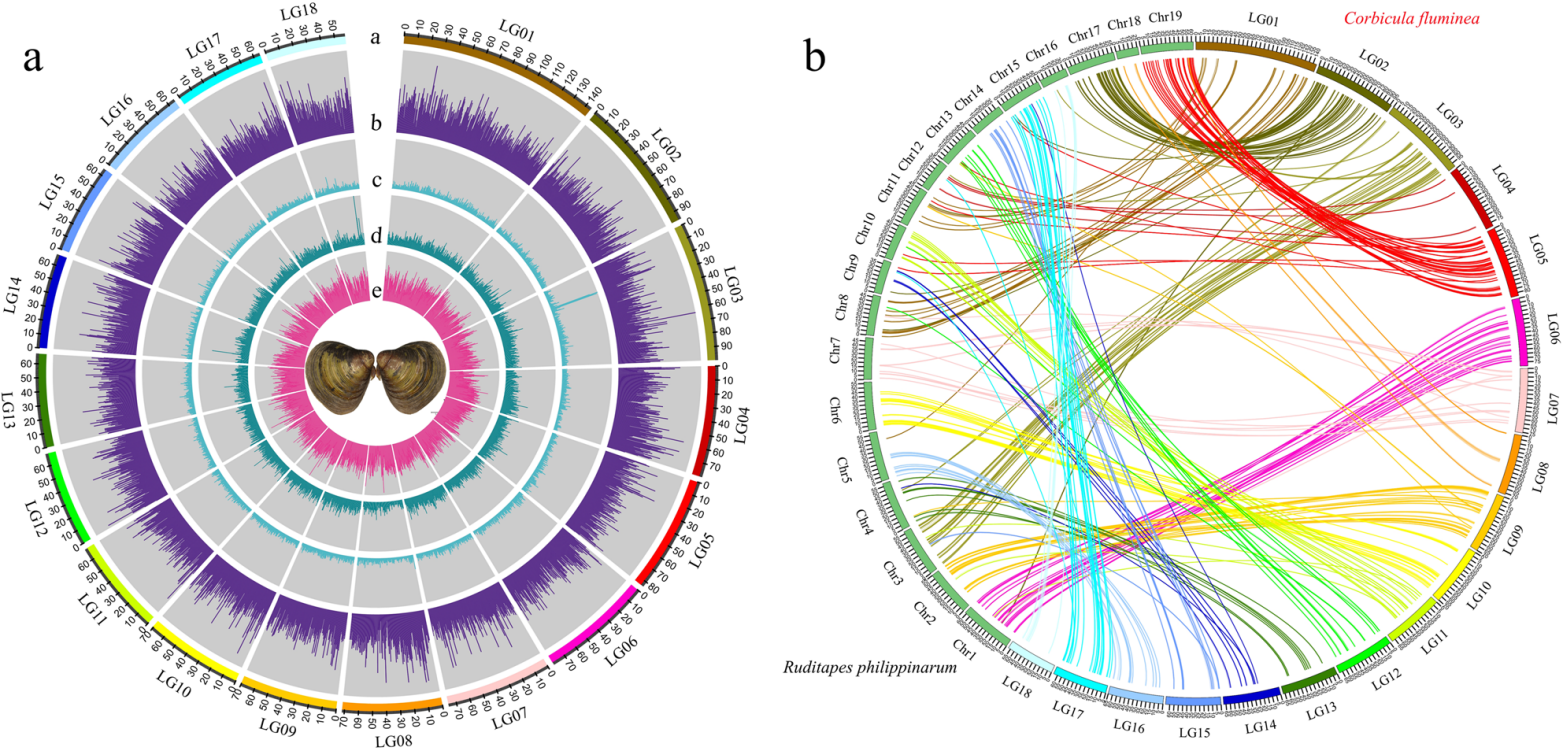
Genome landscape of *Corbicula fluminea* and the syntenic blocks between *C. fluminea and Ruditapes philippinarum*. (**a**) In the middle of the circle are *C. fluminea*. From outer to inner circles: a: marker distribution on 18 chromosomes at the Mb scale; b: LARD distribution on each chromosome; c: PLE distribution on each chromosome; d: gene distribution on each chromosome; e: GC content within a 1-Mb sliding window. (**b**) Syntenic blocks of *C. fluminea* and *R. philippinarum*.

A consensus of the results of all three methods for protein-coding genes prediction was reached, and the final number of non-redundant protein-coding genes was 38,841, with a total length of 0.54 Gb (Table S6). More than 32,591 protein-coding genes (83.91%) were annotated in at least one functional database (Table S7). All genes for each database are annotated in Table S8. Additionally, the Asian Clam gene sets comprised 260,971 exons, and the average gene length was ~ 13.97 kb. The Asian Clam genome contained 3048 pseudogenes, 45 microRNAs, 420 rRNAs, and 3,707 tRNAs (Table S9). Through gene annotation, a clear and comprehensive recognition of the position information of protein-coding genes and non-coding sequences in the genome of the Asian Clam was obtained (Fig. 2a).

### Comparative result of *C. fluminea* and *Ruditapes philippinarum* genomes

We had made statistical analysis on the key indicators of the genome of *C. fluminea* and *R. philippinarum* (Table 1). The *R. philippinarum* genome had a repeat content of 38.29% and a heterozygosity rate of 1.03%. Compared with it, the *C. fluminea* genome had a relatively high repeat content (69.66%) and a high heterozygosity rate (2.41%). The scaffold N50 for *C. fluminea* was 70.62 Mb, whereas that for *R. philippinarum* was 56.47 Mb. The contig N50 of 521.06 Kb for *C. fluminea* was much higher than that of 28.11 kb for *R. philippinarum*. These results suggest that the *C. fluminea* genome, which is assembled on the basis of PacBio reads, Illumina reads, and Hi-C data, is of high quality. Compared with the estimated genome size of *R. philippinarum* (~ 1.32 Gb), that of *C. fluminea* was larger (1.64 Gb). The genome assembly work for *R. philippinarum* eventually produced a genome size of 1.12 Gb, which covered 84.85% of the estimated genome. The *C. fluminea* genome assembled a total of 1.52 Gb of genomic sequences, which covered 92.68% of the estimated genome. The other comparisons, including gene mean length, BUSCO evaluation, and the number of coding genes, etc., showed the genomic characteristics for these two species (Table 1). There were 19 and 18 chromosomes in *R. philippinarum* and *C. fluminea* genomes, respectively. The longest chromosome for *C. fluminea* was the chromosome 01, with a length of 144.27 Mb, whereas the longest chromosome 19 for *R. philippinarum* was only 62.15 Mb (Table S10). The longest chromosome 01 for *C. fluminea* also happened to be the maximum scaffold (144.27 Mb) we assembled. The syntenic analysis generated 244 syntenic blocks between two genomes (Fig. 2b, Table S10). Among that, the most 35 blocks on chromosome 05 of *C. fluminea* were discovered in the genome of *R. philippinarum*, of which 30 blocks occurred on chromosome 19 of *R. philippinarum*. The other relatively high collinearities between *C. fluminea* and *R. philippinarum* genomes were that 26 blocks on chromosome 02 of *C. fluminea* matched the chromosome 17 of *R. philippinarum*; 18 blocks on chromosome 09 of *C. fluminea* matched the chromosome 02 of *R. philippinarum*; 17 blocks on chromosome 06 of *C. fluminea* matched the chromosome 01 of *R. philippinarum*, etc. The chromosome 04 and 08 of *C. fluminea* contained the least blocks, on which was 3 blocks. The blocks on chromosome 06, 10, 13, 16 and 18 of *C. fluminea* individually matched the unique chromosomes in *R. philippinarum* genome.

**Table 1:** Comparative analysis between the genome of *Corbicula fluminea* and the genome of *Ruditapes philippinarum.*

Characteristics	*Corbicula fluminea*	*Ruditapes philippinarum*
Estimate of genome size	1.64 Gb	1.32 Gb
Final assembly genome size	1.52 Gb	1.12 Gb
Contig N50 length	521.06 Kb	28.11 Kb
Maximum contig length	3.17 Mb	249.66 Kb
Scaffold N50 length	70.62 Mb	5.65 Mb
Maximum scaffold length	144.27 Mb	20.46 Mb
Average chromosome length	77.68 Mb	48.66 Mb
Maximum chromosome length	144.27 Mb	62.15 Mb
Minimum chromosome length	57.93 Mb	25.99 Mb
Heterozygosity rate	2.41%	1.03%
Repeat percentage	69.66%	38.29%
Total protein-coding genes	38,841	27,652
Average gene length	13.97 Kb	12.87 Kb
BUSCO assessment	C:86.6% [S:73.0%, D:13.6%], F:1.5%, M:11.9%, n:5295	C:91.0% [S:89.3%, D:1.7%], F:3.9%, M:5.1%, n:978

### Analysis of protein families

**Figure 3:**
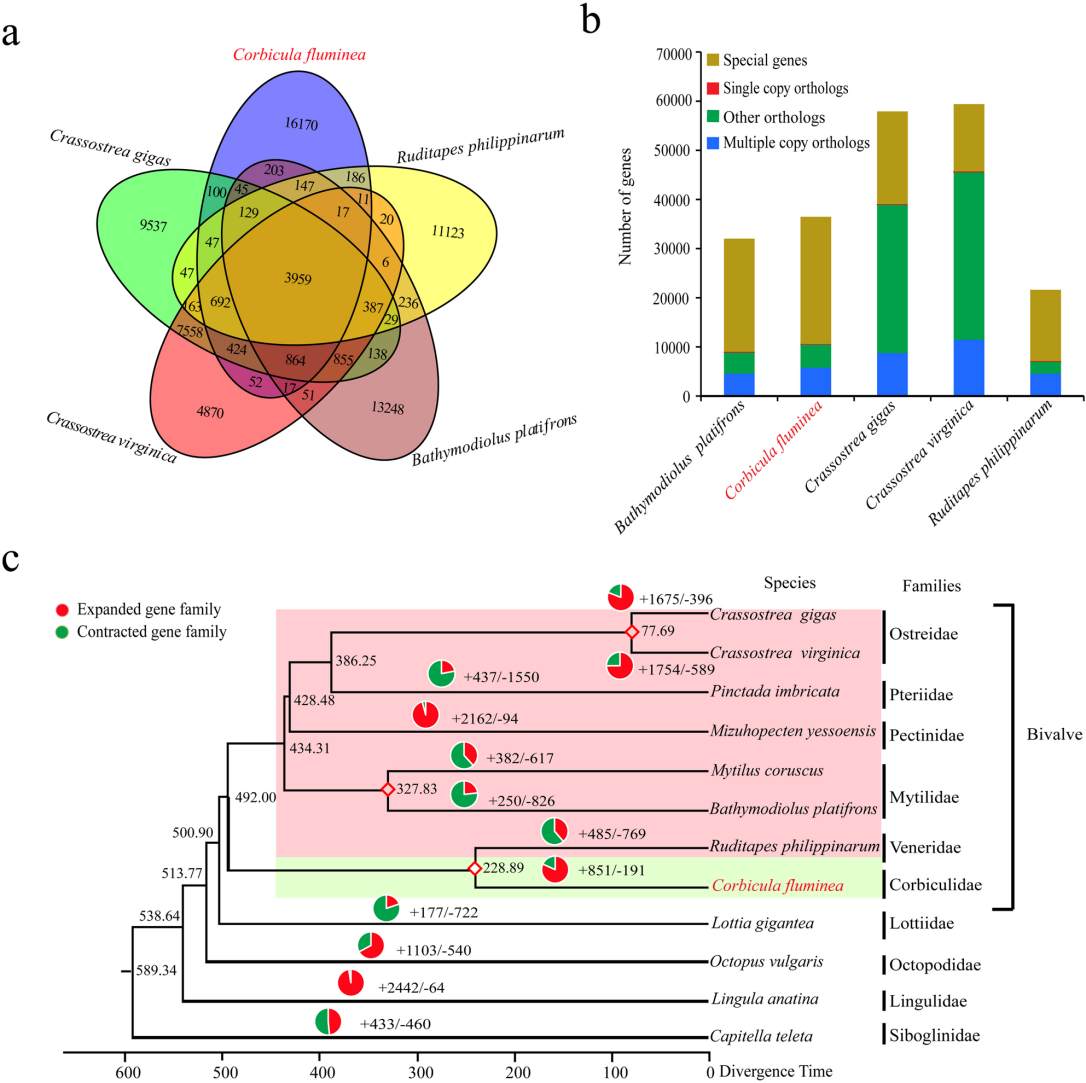
The comparative genomic analysis of *Corbicula fluminea* and other species. (**a**) Venn diagram of gene families between *C. fluminea* and *Crassostrea gigas*, *Ruditapes philippinarum*, *Bathymodiolus platifrons*, and *Crassostrea virginica*. (**b**) Distribution of multiple-copy orthologs, other orthologs, single-copy orthologs, and unique genes in *C. fluminea* and the above four species. (**c**) Phylogenetic tree, divergence time, and profiles of gene families that underwent expansion and contraction in 12 species.

Gene family analysis identified a total of 71,331 gene families among five species of bivalves (Table S11), and we discovered 23,063 gene families clustered by 38,841 protein-coding genes in the Asian Clam genome. Compared with the genome of *R. philippinarum*, *Crassostrea gigas*, *Crassostrea virginica*, and* Bathymodiolus platifrons*, the *C. fluminea* genome had 16,170 specific gene families (Fig. 3a). Additionally, Single-copy orthologs, multiple copy orthologs, other orthologs, and unique genes were identified in the all-to-all BLASTP analysis of entries for the reference genomes. The five bivalve species shared 146 single-copy orthologs, and the Asian Clam genome contained 25,878 unique genes (Fig. 3b, Table S12).

### Phylogenetic and gene family expansion analysis

The phylogenetic relationship between *C. fluminea* and other representative species was estimated based on single-copy orthologs. Three time points for the most recent common ancestor (MRCA) were estimated by TimeTree. The differentiation time of *Crassostrea gigas* and *Crassostrea virginica* was 72.9 (63.2–82.7) million years ago (Mya)^27^; that of *B. platifrons* and *Mytilus coruscus* was 387 (308–481) Mya^28^; that of *C. fluminea* and *R. philippinarum* was 244 (114–280) Mya^29^. We utilized these time of MRCA to calibrate the phylogenetic tree, resulting in the phylogenetic tree constructed by eight bivalves and four other molluscs species (Fig. 3c). As shown, all bivalves were clustered together, especially those belonging to the same family/order. The phylogenetic tree showed that *C. fluminea* and its closest relative, *R. philippinarum*, diverged at an early stage of ~ 228.89 million years ago. The ancestors of *C. fluminea* and *R. philippinarum,* diverged from the common ancestors of other six marine bivalves (family Mytilidae represented by *B. platifrons* and *Mytilus coruscus*; family Ostreidae represented by *Crassostrea gigas* and *Crassostrea virginica;* family Pteriidae represented by *Pinctada imbricata;* family Pectinidae represented by *Mizuhopecten yessoensis*), ~ 492.00 million years ago.

Combining the phylogenetic relationships, gene family evolution was calculated by comparing the differences between ancestors and *C. fluminea*. This analysis resulted in 851 gene families being significantly expanded (P < 0.05) and 191 gene families being significantly contracted (P < 0.05) in the Asian Clam genome (Fig. 3c, Table S13). The 851 expanded gene families were clustered by 9,967 functional genes (Table S14). The functional enrichment analysis on GO and KEGG of those expanded genes identified 325 significantly enriched (q-value < 0.01) GO terms (Table S15) and 19 significantly enriched (q-value < 0.01) KEGG pathways (Fig. S2, Table S16). Among the significantly enriched KEGG pathways, we found taurine and hypotaurine metabolisms were significantly enriched.

### MITF gene family analysis

**Figure 4:**
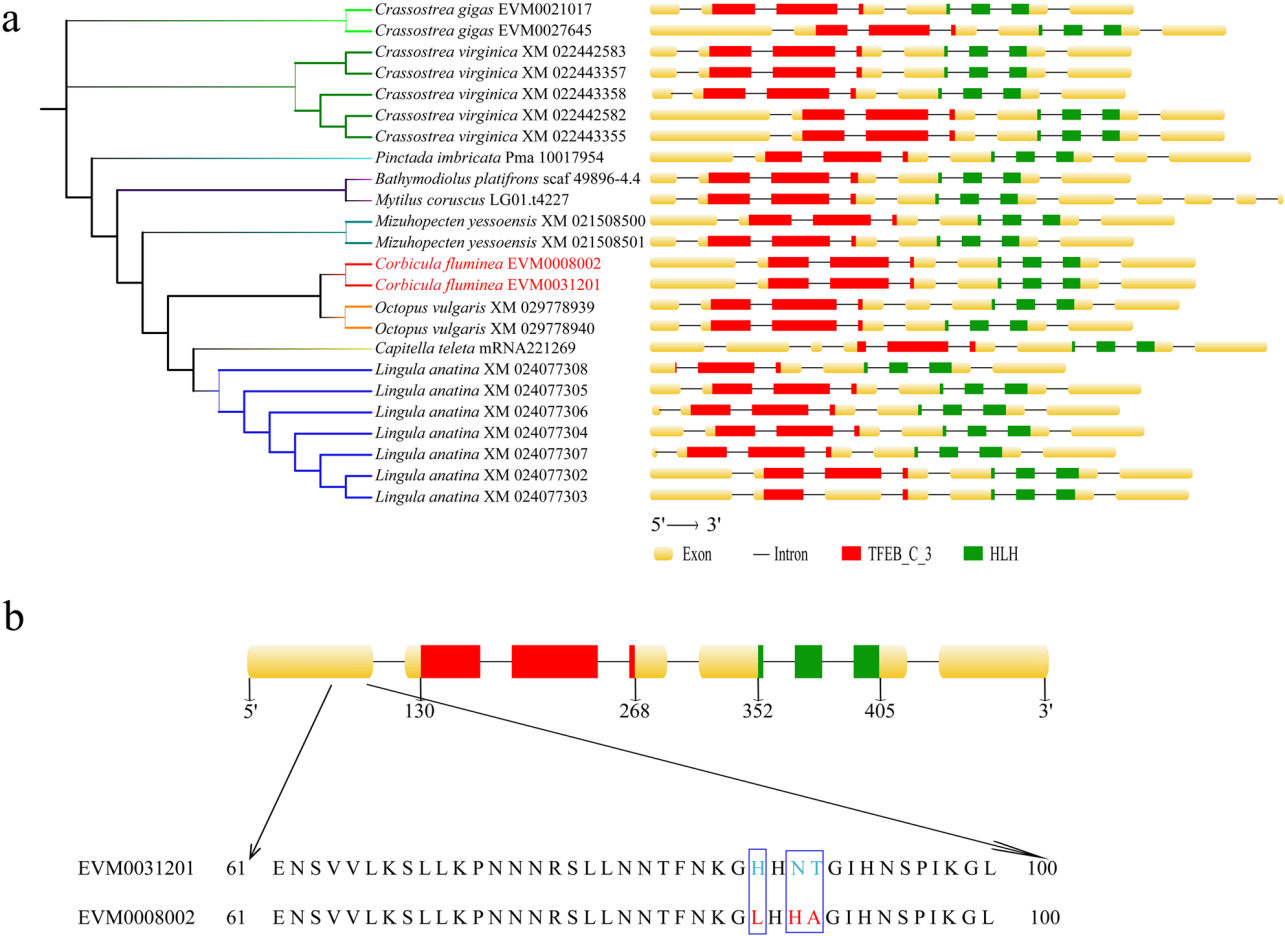
The analysis of MITF gene family. (**a**) The members of MITF family in *Corbicula fluminea* and other species. (**b**) The Commonalities and differences for MITF members in *C. fluminea.*

The genic tree comprising all MITF family genes was successfully constructed using MUSCLE (Fig. 4a). Most species possessed one or two MITF members, while *Lottia gigantea* lost MITF members. *Crassostrea virginica* and *L. anatine* possessed five and seven MITF members, respectively (Table S17). This result coincides with the result of the above gene family evolution analysis, which showed the MITF gene family expanded in *Crassostrea virginica* and *Lingula anatina*, and contracted in *Lottia gigantea* (Table S18). The genic tree also showed that MITF members originated from the same species were clustered at the nearest genetic distance. MITF members from the same families (family Mytilidae represented by *B. platifrons* and *Mytilus coruscus*, family Ostreidae represented by *Crassostrea gigas* and *Crassostrea virginica*), were clustered more together. The clustering relationships of MITF gene family were similar to those shown by the phylogenetic tree of single-copy orthologs. This finding indirectly corroborates the reliability of the phylogenetic relationship analysis.

In this study, we detected two members from the Asian Clam genome, namely EVM0008002 and EVM0031201, which were identified as MITF genes. Both genes contained an N-terminal domain TFEB_C_3 and a highly conserved functional domain HLH. The EVM0008002 was located at 47.05–47.08 Mb on chromosome 10, with a length of 28,761 bp, and encoded 533 amino acids. The position of EVM0031201 was close to that of EVM0008002, and it was also located on chromosome 10. The EVM0031201 was located at 46.99–47.02 Mb, with a length of 29,767 bp, and it encoded 533 amino acids, too. Both EVM0008002 and EVM0031201 contained 8 exons that comprising 533 amino acids, and 7 introns. The domain TFEB_C_3 of them started with 130 amino acids and ended with 268 amino acids, and was accompanied by 3 exons. The domain HLH of them started with 352 amino acids and ended with 405 amino acids, and was accompanied by 3 exons, too (Fig. 4b). Among 533 amino acids, the types and sequences of 530 amino acids for these two genes were consistent, only three amino acids showed the differences. The three differences of amino acids were located at position of 87, 89, and 90, respectively. Specifically, the amino acids of EVM0008002 at position of 87, 89, and 90, were Leucine (L), Histidine (H), and Alanine (A), respectively. The amino acids of EVM0031201 at position of 87, 89, and 90, were Histidine (H), Asparagine (N), and Threonine (T), respectively (Fig. 4b).

### NLRP gene family analysis

**Figure 5:**
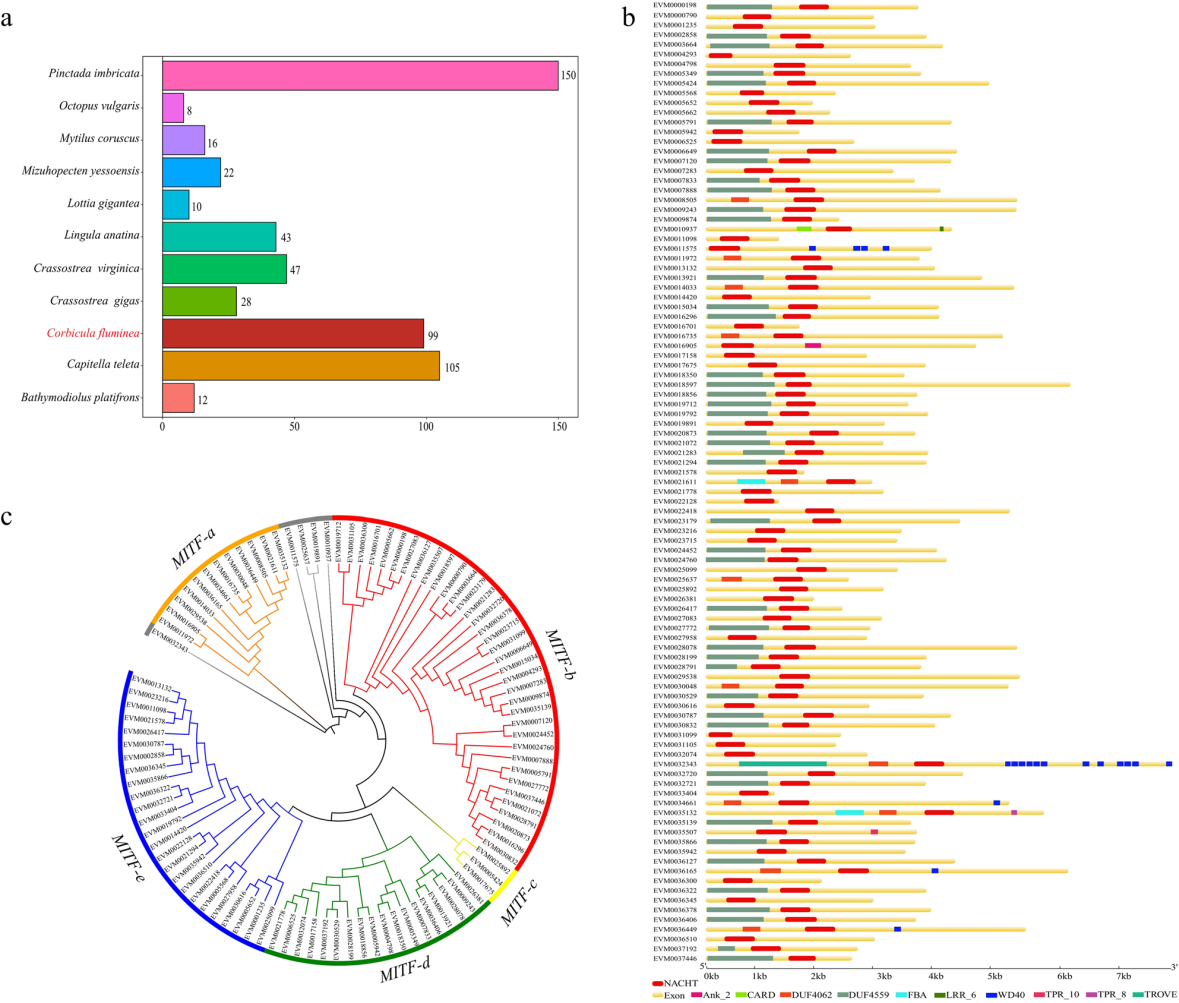
The analysis of NLRP gene family. (**a**) The number of NLRP members in *Corbicula fluminea* and other species. (**b**) The domains of NLRP members in *C. fluminea.* (**c**) NLRP members in *C. fluminea* were divided into five subfamilies, namely Subfamily (a–e).

NLRP (Nucleotide-binding oligomerization domain, Leucine rich Repeat and Pyrin domain containing Proteins) is well known for its roles in apoptosis and inflammation. Among all the species involved in the evolutionary analysis, the number of NLRP members in *C. fluminea* (99) was more than that of most species, except *P. imbricata* (150) and *Capitella teleta* (105) (Fig. 5a). Specifically, the number of NLRP members in *C. fluminea* was more than that shown in *B. platifrons* (12), *Mytilus coruscus* (16), *Mizuhopecten yessoensis* (22)*, Crassostrea gigas* (28), *Crassostrea virginica* (47). Additionally, we analyzed the domain NACHT of *C. fluminea* (99) in the table of the expanded gene families in *C. fluminea* (Table S14), which was significantly expanding compared to its ancestors (10). Among the 99 NLRP members in *C. fluminea* genome, 45 members possessed domain DUF4559, 12 members possessed domain DUF4062, and 5 members possessed the domain WD40, etc. (Fig. 5b, Table S19). Meanwhile, we found that all five members (EVM0034661, EVM0036165, EVM0036449, EVM0010937 and EVM0021611) contained 3 domains, and two members (EVM0032343 and EVM0035132) contained 4 domains. The NLRP members of *C. fluminea* grouped into five subfamilies (subfamily a–e) (Fig. 5c). Subfamily a owned 12 members clustered by the same or similar protein domains, as the same as subfamily b to e possessed 36, 3, 18, and 25 members, respectively (Table S20). Five of 99 members did not cluster into any subfamily.

## Methods

### Sample collection and DNA isolation

Fresh Asian Clam (*C. fluminea*) samples were collected from Hongze Lake (118.18 E, 33.22N), Jiangsu, China. Healthy and disease-free individuals of *C. fluminea* were selected as sequencing individuals. After the physical removal of shells and gut content, the whole soft bodies were immediately transferred into liquid nitrogen. High-quality genomic DNA was extracted from the body of Asian Clam using a DNeasyR Blood& Tissue Kit (Qiagen, Hilden, Germany). The DNA quality was measured with Qubit 3.0 (Invitrogen, Carlsbad, CA, USA) and was checked using 1% agarose gel electrophoresis.

### Library preparation and sequencing

#### Whole-genome shotgun sequencing

The libraries of short insert size (350 bp) for Illumina were constructed according to the manufacturer’s standard PCR-free protocol (Illumina) and sequenced on an Illumina HiSeq X Ten platform (Illumina, Inc., San Diego, CA, USA) using the paired-end 150 (PE150) strategy. Six Illumina libraries were used to produce data for survey analysis and PacBio error correction.

#### Pacific biosciences technologies

Approximately 30 μg of genomic DNA was used to construct PacBio libraries by shearing into ~ 20 kb targeted size fragments with Blue Pippin (Sage Science, Beverly, MA, USA). Then, the qualified libraries were prepared for single-molecule real-time (SMRT) genome sequencing using S/P2-C2 sequencing chemistry on the PacBio Sequel II platform (PacBio, Pacific Biosciences, USA). Two PacBio libraries generated data for genome assembly.

#### Hi-C technologies

DNA was extracted from the whole body with the gut removed, and it was cross-linked in situ using formaldehyde with a final concentration of 2% and homogenized with tissue lysis by the restriction enzyme HindIII. The libraries for Hi-C with insert sizes of 300–700 bp were sequenced on an Illumina HiSe q X Ten platform (Illumina, SanDiego, CA, USA). Two Hi-C libraries generated data for chromosomal building.

#### Transcriptome sequencing

Using TRIzol (Thermo Fisher, USA), the RNA was extracted from the whole body with the gut removed, and the libraries were generated using a NEBNext Ultra RNA Library Prep Kit for Illumina (NEB, USA) following the instruction manual. The data was used to alignment to the assembled genome for prediction of coding genes.

### Genome estimation

Illumina reads were aligned to the Nucleotide Sequence Database (NT) using BLAST (version 2.2.31)^30^ with the parameter of E-value = 1e^−05^ for contamination verification. Then, Illumina data were filtered and corrected by Fastp (version 0.19.3)^31^, followed by k-mer analysis to estimate the genomic features. In this study, we plotted the 21-mer depth distribution (k = 21) to estimate the genome size, heterozygosity, and repeats using Jellyfish (version 2)^32^. Genome size estimation was implemented by the formula G = N21-mer (total number of k-mers)/D 21-mer (k-mer depth of the main peak). The repetitive content was accumulated from where the depth of k-mer was more than two times of the main peak, and the heterozygosity were estimated at where the depth was half of the main peak.

### Denovo assembly

Using the long single molecular reads from PacBio, the pipelines of workflow were as follows in the genome assemblies. Firstly, the clean data from PacBio were subjected to error correction using Canu (version 1.5)^33^ with the parameter of error correct coverage = 60. Subsequently, the outputs were piped into the workflow of SMART denovo (version 1.0)^34^, and the genomic contigs were automatically generated with the parameters of J = 5000, A = 1000, and r = 0.95. Finally, the preliminary assembly was polished three times by Racon (version 1.32)^35^, resulting in the first correction being successfully realized. Illumina reads specifically for genome estimation were prepared for the second correction, and this round of correction could solve the high error rate of the third generation sequencing. The third round of correction was implemented by Pilon (version 1.22)^36^, and the error correction was run for three times.

### Hi-C scaffolding

The contigs generated by the preliminary genome assembly required filling of gaps and anchoring on the putative chromosomes. The initial contigs were piped into the Hi-C assembly workflow, and the signals of chromatin interactions were captured to construct chromosomes. In brief, the putative Hi-C junctions were aligned by the unique mapped read pairs using BWA-MEM (version 0.7.10-r789)^37^. The paired reads uniquely mapped to the assembly were called the valid interaction pairs, and they were used for the Hi-C scaffolding. Other invalid reads included reads of self-ligation and non-ligation; dangling ends were filtered out using HiC-Pro (version 2.10.0)^38^. The Hi-C reassembly broke the contigs into 50 kb fragments, and the regions that were mismatched to the initial assembly or could not be restored were listed as candidate error areas. The genome was subjected to a final round of error correction, and the gaps were filled during this round. The reassembled and corrected contigs were divided into ordered, oriented, and anchored groups by LACHESIS^39^ with the parameters CLUSTER_MIN_RE_SITES = 33; CLUSTER_MAX_LINK_DENSITY = 2; CLUSTER_NONINFORMATIVE_RATIO = 2; ORDER_MIN_N_RES_IN_TRUN = 29, and ORDER_MIN_N_RES_IN_SHREDS = 29, automatically resulting in putative chromosomes. The gaps generated during the Hi-C assembly were refilled using LR GapCloser (version 1.1)^40^.

### Genome quality evaluation

The genome of *C. fluminea* was aligned to the Mollusca database (OrthoDB10) comprising 5,295 conservative core genes by BUSCO (version 3.0)^41^. The CEGMA Database comprising 458 conserved core genes of eukaryotes was searched in the same way using CEGMA (version 2.5)^42^. The Illumina short-read alignments mapped to the assembled genome of the Asian Clam using BWA-MEM (version 0.7.10-r789)^37^.

### Repeats analysis

There are two main types of repeats, retrotransposons (Class I in our analysis) and transposons (Class II in our analysis).We constructed a specific repeats database for repeat prediction using LTR-FINDER (version 1.05)^43^ and RepeatScout (version 1.0.5)^44^, followed by the identification and classification for repeats by PASTEClassifer (version 1.0)^45^. The species-specific repeats library for the Asian Clam genome was successfully generated by aggregating our prediction and Repbase (19.06)^46^. LTR characteristics for the clam were processed by RepeatMasker (version 4.0.6)^47^.

### Genome annotation

#### Gene annotation

We utilized de novo-, homology-, and transcriptome-based methods to predict protein-coding genes. Five tools employed were Genscan (verson3.1)^48^, Augustus (version 3.1)^49^, GlimmerHMM (version 3.0.4)^50^, GeneID (version 1.4)^51^, and SNAP (version 2006-07-28)^52^; these were used for prediction de novo. Protein sequences from four representative species (*Danio rerio*, *Crassostrea gigas*, *Crassostrea virginica*, and *Mizuhopecten yessoensis*) were aligned to the genome scaffolds of Asian Clam to perform homology-based prediction by GeMoMa (version 1.3.1)^53^. Transcriptome data were mapped to the genomic sequences; Hisat (version 2.0.4)^54^ and Stringtie (version 1.2.3)^55^ were used to assemble and dissect functional genes. TransDecoder (version 2.0) (http://transdecoder.github.io) and GeneMarkS-T (version 5.1)^56^ were used for transcriptome-based prediction. Finally, the above methods were integrated into non-redundant protein-coding gene sets by EVM (version 1.1.1)^57^ and PASA (version 2.0.2)^58^.

#### Non coding gene annotation

The other genome features, including pseudogenes and non-coding RNAs, were identified by referring to the miRbase database (version 21.0)^59^ and Rfam (version 13.0)^60^. In the process of searching for putative pseudogenes, candidates were assessed based on the premature stop codons or frame shift mutations in the gene structure using GenBlastA (version 1.0.4)^61^. The identification of transfer RNA (tRNA) was performed by tRNAscan-SE (version 1.3.1)^62^. MicroRNA and ribosomal RNA (rRNA) were identified by Infernal (version 1.1)^63^.

#### Gene function annotation

The protein-coding genes were subject to functional annotation by aligning to the EuKaryotic Orthologous Groups (KOG)^64^, Kyoto Encyclopedia of Genes and Genomes (KEGG)^65^, TrEMBL^66^, Swiss-Prot^66^, and Non-redundant (Nr) databases^67^ using BLAST (version 2.2.31)^30^ with a maximal E-value of 1e−05. Kyoto Encyclopedia of Genes and Genomes (KEGG) pathway annotations and Gene ontology (GO)^68^ terms were assigned to identify gene functions using Blast2GO (version 4.1)^69^.

The position information of protein-coding genes and non-coding sequences distributed on different chromosomes in the genome of the Asian Clam using Circos (http://circos.ca/software/download/).

### Comparative analysis of *C. fluminea* and *R. philippinarum* genomes

The genome data of *R. philippinarum* (10.1016/j.isci.2019.08.049, 2019) that is also belonging to the order Veneroida was used to conduct the comparative analysis with the *C. fluminea* genome. The process of the comparison included genome size, assembly index, evaluation and collinearity, which was helpful to better understand the genome of *C. fluminea*. For collinearity analysis, we compared the *C. fluminea* genome with the genome of *R. philippinarum* using MUMmer (http://mummer.sourceforge.net), with the parameter l = 10,000. The genomes of *C. fluminea* and *R. philippinarum* were subjected to a synteny analysis to show the connections and syntenic blocks using BLASTP (E < 1e−05)^30^, and the visual graphics were generated by MCScan [https://github.com/tanghaibao/jcvi/wiki/MCscan—(Python-version)]. Each syntenic block comprised at least five sequential genes, which were all distributed in two genomes.

### Gene family identification

Protein data from *C. fluminea* and other representative species (all Bivalve species and some mollusks with assembly and annotation that could be found in NCBI or other databases), including *Capitella teleta*, *Lingula anatina*, *Octopus vulgaris*, *Lottia gigantea*, *R. philippinarum*, *Crassostrea gigas*, *Crassostrea virginica*, *P. imbricata*, *Mizuhopecten yessoensis*, *Mytilus coruscus*, and *Bathymodiolus platifrons*, were retrieved in the corresponding databases and aligned using BLAST (version 2.2.31, https://ftp.ncbi.nlm.nih.gov/blast/executables/blast+/LATEST/)^30^ with a maximum e-value of 1e−5. Proteins with sequence lengths > 100 amino acids were searched against the Pfam (https://pfam.xfam.org) database by Pfam scan^70^. The domain of gene feature was made by the Gene Structure Display Server -GSDS (version2.0)^71^. Protein sequences were clustered using CD-HIT^72^, with a length difference cutoff of 0.7, and finally concatenated to a single fasta file. The ortholog groups for gene families were generally clustered using OrthoMCL (version 2.0.9)^73^. The R package (version 4.1.0, https://mirrors.bfsu.edu.cn/CRAN/) was used to generate the column chart. Four selected bivalves (*R. philippinarum*, *Crassostrea gigas*, *Crassostrea virginica*, and *B. platifrons*) and *C. fluminea* were grouped together to conduct the analysis for gene family characteristics, and the venn was generated by the R package (version 4.1.0, https://mirrors.bfsu.edu.cn/CRAN/).

### Phylogenetic tree reconstruction and divergence time estimation

The single-copy orthologs from all involved species were statistically analyzed using the longest transcripts for each gene. The single-copy orthologous genes shared by the above 12 species (including *C. fluminea*) were aligned using MUSCLE (version 3.8.31)^74^. The super-alignment of nucleotide sequences provided a reference tree topology using PhyML (version 3.3)^75^. The divergence times among species were roughly estimated by the MCMC Tree program of the PAML package (version 4.7a)^76^ with the approximate likelihood calculation method. We utilized molecular clock data from the TimeTree (http://www.timetree.org/)^77^ database as the calibration times. The phylogeny tree was optimized by iTOL (version 6 https://itol.embl.de/).

### Gene family evolutionary analysis

According to divergence times and phylogenetic relationships, CAFÉ (version 4.2)^78^ was used to analyze gene family evolution. The gene family expansion and contraction were analyzed by comparing the differences between the ancestor and involved species. The expanded family genes for *C. fluminea* were extracted and aligned to the functional enrichment on GO and KEGG to detect their functions.

### Prediction of specific protein domains

Pfam database provided protein domains, and the specific proteins with sequence lengths > 100 amino acids, were searched against it for specific gene families analysis. GSDS (version2.0) and R package (version 4.1.0) was used to generate the visual gene feature and the column chart, respectively. The MITF gene family consisted of three domains, namely TFEB, TFEC, and TFE3^79^. In this study, we utilized protein-coding sequences from the representative species to analyze the members of MITF gene family, especially the structure and amino acid composition of members in the *C. fluminea* genome. The core domain of NALP family was NACHT^80^, which was used to analyze the structure and distribution of NALP family members in *C. fluminea* genome.

## Discussion

In this study, we assembled a chromosome-level Asian Clam genome using a combination of PacBio and Hi-C technology. Generally, a complex genome is defined as a heterozygosity ratio greater than 0.8% and a repeat ratio greater than 60%. The high repeats (69.66%) and heterozygosity rate (2.41%) of *C. fluminea* genome bring great difficulties to assembly, we still assembled and obtained a high-quality and chromosomal genome. The 1.52 Gb of genome data distributed across 18 chromosomes, with a contig N50 of 521.06 Kb and a scaffold N50 of 70.62 Mb. The scaffolding process for the Asian Clam genome showed a high level of efficiency (more than 99% genomic sequences and more than 97% contigs were located on chromosomes). The 18 chromosomes of *C. fluminea* covered 92.68% of the whole genome, and the longest chromosome 01 was 144.27 Mb. These data results are strong evidence of our ultra-high quality genome.

In present study, the phylogenetic relationship suggested that the ancestors of *C. fluminea* and its closest relative *R. philippinarum* diverged from the common ancestors of other six bivalves, ~ 492.00 million years ago. It is consistent with the origin time of Heterodonta from the Paleozoic^81,82^. The genetic distance between the two species and other marine bivalves is relatively far. However, despite *C. fluminea* and *R. philippinarum* share over 240 syntenic genome blocks, there are still great habitat and adaptation differences between them. The majority of *C. fluminea* is living in typical freshwater ecosystem, while the brackish water species *R. philippinarum* is mainly distributed in the coastal area^83^. The phylogenetic relationship showed that *C. fluminea* and *R. philippinarum* diverged at an early stage of ~ 228.89 million years, coinciding with the divergency event of Veneroida occurring in the Mesozoic and Cenozoic eras^84^. This evidence suggests that as a freshwater bivalve, *C. fluminea* had been diverged from other bivalves million years ago. A long-term divergency and evolutional process resulted in the unique survival mechanism or environmental adaptation of the Asia Clam. Thus, the ancestors of *C. fluminea* might have invaded and migrated to freshwater from the ocean since millions of years ago, and they have evolved to fill various freshwater habitat.

On account of short sexual maturity time, rapid growth, short life cycle and planktonic veliger stage, *C. fluminea* has strong diffusion ability^85^, and it is considered as an alien species in America and Europe^11–13^. The strong reproductive capacity and a powerful immune system might be bound to play an important role. In this study, we identified two gene families, MITF and NLRP, which were respectively related to the immune and reproductive adaptability of *C. fluminea.* It has been reported that microphthalmia-associated transcription factor (MITF) plays an important role in immune defense and shell color formation in molluscs^86,87^. We identified two MITF genes (EVM0008002 and EVM0031201) in the Asian Clam genome. They both encoded 533 amino acids, only three of which were different. These two genes were located on chromosome 10, and their physical distance was very close. Specifically, EVM0031201 was located at 46.99–47.02 Mb, and EVM0008002 was located at 47.05–47.08 Mb. The EVM0031201 and EVM0008002 were so close to each other, which may be a duplication of the genome region, and this duplication may include one or more genes. Except for functions in apoptosis and inflammation, several NLRPs have been indicated as being involved in reproduction as well^88^. The 99 members of NLRP family in *C. fluminea* genome were significantly more than that of most of the candidate species, and the NLRP gene family was significantly expanded comparing to its ancestors, with 10 NLRP members. We infer the expansion of NLRP family may be related to the strong reproductive function of *C. fluminea*. The genomic information presented in our analysis will help to better understand, develop, and improve *C. fluminea* as well as establish a strong foundation for genome-assisted breeding programs in the future.

## Supplementary Information

Supplementary Information 1.

Supplementary Figure S1.

Supplementary Figure S2.

Supplementary Table S8.

Supplementary Table S9.

Supplementary Table S10.

Supplementary Table S11.

Supplementary Table S13.

Supplementary Table S14.

Supplementary Table S15.

Supplementary Table S16.

Supplementary Table S17.

Supplementary Table S18.

Supplementary Table S19.

Supplementary Table S20.

## Data Availability

Raw sequencing reads for PacBio and Illumina are available at GenBank as BioProject PRJNA657911. Raw sequencing data (Illumina, PacBio, and Hi-C data) have been deposited in the SRA (Sequence Read Archive) database as SUB7507164. The data including assembly and annotation that supported the findings of this study have been deposited in the in the FigShare database, (10.6084/m9.figshare.12805886.v1).
