# Supplementary material for: Dissecting the chromosome-level genome of the Asian Clam (Corbicula fluminea)
Source: Sci Rep. 2021 Jul 22;11:15021. doi: 10.1038/s41598-021-94545-2 (PMC8298618; doi:10.1038/s41598-021-94545-2)
Supplement: Supplementary file 1 — Supplementary Information 1. [file 41598_2021_94545_MOESM1_ESM.docx]

**Additional files**

Supplementary Figure S1. Frequency distribution of the 21-mer graph analysis used to estimate the features of *Corbicula fluminea* genome.

Supplementary Figure S2. KEGG enrichment analysis for expanded family genes in *Corbicula fluminea*.

Supplementary Table S1.Statistics of the sequencing data.

Supplementary Table S2.Statistics of the different types of Hi-C reads.

Supplementary Table S3.Summary of the Hi-C assembly.

Supplementary Table S4.Summary of the assessment of genome assembly.

Supplementary Table S5.Statistics of the repeated sequences.

Supplementary Table S6.Summary of the gene prediction results.

Supplementary Table S7.Statistics of gene annotation to different databases.

Supplementary Table S8.Integrated lists of gene annotation for *Corbicula fluminea* genome.

Supplementary Table S9. The family consisting of miRNAs, rRNAs and tRNAs in *Corbicula fluminea.*

Supplementary Table S10.Chromosomes and syntenic blocks between *Corbicula fluminea* genome and *Ruditapes philippinarum* genome.

Supplementary Table S11. The identification of gene families among five bivalves

Supplementary Table S12. Summary of gene features in five bivalves.

Supplementary Table S13.The expanded and contracted gene families in *Corbicula fluminea.*

Supplementary Table S14.The expanded family genes in *Corbicula fluminea.*

Supplementary Table S15.Significantly enriched GO terms for expanded family genes in *Corbicula fluminea.*

Supplementary Table S16.Significantly enriched KEGG pathways for expanded family genes in *Corbicula fluminea.*

Supplementary Table S17.MITF genes between *Corbicula fluminea* and other representative species.

Supplementary Table S18.The expanded and contracted gene families in representative species.

Supplementary Table S19.The domains for NLRP family members in *Corbicula fluminea.*

Supplementary Table S20. The 99 NLRP family members in *Corbicula fluminea* were grouped into subfamily a-e.

**Table S1:** Statistics of the sequencing data

| Types | Sequencing  platform | Library size | Number of library | Clean data  (Gb) | Coverage  (×) † |
| --- | --- | --- | --- | --- | --- |
| Illumina | Illumina HiSeq X | 350 bp | 6 | 252.77 | 154.13 |
| PacBio | PacBio Sequel II | 20 kb | 2 | 293.72 | 193.40 |
| Hi-C | Illumina HiSeq X | 300–700 bp | 2 | 233.26 |  |
| Transcriptome | Illumina HiSeq X | 350 bp | 1 | 8.18 | - |

**Table S2:** Statistics of the different types of Hi-C reads

| Mapping type | | Ratio to total read pairs (%) | Ratio to unique mapped read pairs (%) |
| --- | --- | --- | --- |
| Total read pairs | | 780,870,254 (100) | - |
| Mapped read pairs | | 571,597,025 (73.20) | - |
| Unique mapped read pairs | | 188,834,895 (24.18) | 188,834,895 (100) |
| Valid interaction pairs | | 116,652,252 (14.94) | 116,652,252 (61.77) |
| Invalid interaction pairs | Dangling end pairs | 72,182,643 (9.24) | 32,181,456 (17.04) |
|  | Re-ligation pairs |  | 13,552,964 (7.18) |
|  | Self-cycle pairs |  | 1,289,195 (0.68) |
|  | Dumped pairs |  | 25,159,028 (13.32) |

**Table S3:** Summary of the Hi-C assembly

| Group | Cluster number | Cluster length (bp) | Order number | Order length (bp) |
| --- | --- | --- | --- | --- |
| Chr01 | 470 | 153,803,806 | 371 | 144,229,830 |
| Chr02 | 313 | 107,196,770 | 231 | 96,729,369 |
| Chr03 | 326 | 105,879,025 | 262 | 99,673,625 |
| Chr04 | 353 | 89,177,396 | 255 | 79,719,718 |
| Chr05 | 190 | 87,093,095 | 156 | 83,870,643 |
| Chr06 | 249 | 85,721,425 | 191 | 79,888,130 |
| Chr07 | 272 | 82,224,449 | 201 | 75,738,333 |
| Chr08 | 339 | 80,407,287 | 252 | 71,696,766 |
| Chr09 | 236 | 76,323,934 | 172 | 70,155,845 |
| Chr10 | 206 | 75,434,972 | 158 | 70,608,841 |
| Chr11 | 203 | 73,470,129 | 156 | 68,962,617 |
| Chr12 | 227 | 73,804,105 | 181 | 68,909,514 |
| Chr13 | 208 | 72,126,331 | 155 | 67,123,217 |
| Chr14 | 225 | 72,953,836 | 170 | 67,734,440 |
| Chr15 | 218 | 69,882,476 | 166 | 64,959,859 |
| Chr16 | 177 | 68,966,225 | 136 | 65,096,897 |
| Chr17 | 200 | 69,709,078 | 148 | 64,804,978 |
| Chr18 | 209 | 61,886,338 | 170 | 57,909,238 |
| Total (Ratio %) | 4,621 (97.74) | 1,506,060,677 (99.17) | 3,531 (76.41) | 1,397,811,860 (92.81) |

**Table S4:** Summary of the assessment of genome assembly

| Parameter | BUSCO groups (%) | CEGMA groups (%) | Illumina reads ratio (%) |
| --- | --- | --- | --- |
| Total BUSCOs | 5295 (100) | - | - |
| Complete BUSCOs | 4588 (86.65) | - | - |
| Complete and single-copy BUSCOs | 3867 (73.03) | - | - |
| Complete and duplicated BUSCOs | 721 (13.62) | - | - |
| Fragmented BUSCOs | 79 (1.49) | - | - |
| Missing BUSCOs | 628 (11.86) | - | - |
| Total CEGMA | - | 458 (100) | - |
| Searched CEGMA | - | 423 (92.36) | - |
| Number of 248 highly conserved | - | 205 (82.66) | - |
| Total reads | - | - | 1,664,680,996 (100) |
| Mapped reads | - | - | 1,622,200,797 (97.45) |
| Mapped reads pairs | - | - | 1,469,907,318 (88.30) |

**Table S5:** Statistics of the repeated sequences

| Type | Number | Length（bp） | Percentage (%) |
| --- | --- | --- | --- |
| ClassI/DIRS | 24,688 | 10,566,441 | 1.00 |
| ClassI/LARD | 2,515,771 | 608,849,833 | 57.54 |
| ClassI/LINE | 235,902 | 74,779,768 | 7.07 |
| ClassI/LTR/Copia | 24,935 | 6,273,557 | 0.59 |
| ClassI/LTR/Gypsy | 106,541 | 49,198,634 | 4.65 |
| ClassI/PLE | 445,851 | 130,983,883 | 12.38 |
| ClassI/SINE | 102 | 69,215 | 0.01 |
| ClassI/TRIM | 25,844 | 14,743,872 | 1.39 |
| ClassII/Crypton | 21,742 | 8,053,927 | 0.76 |
| ClassII/Helitron | 179,095 | 54,933,929 | 5.19 |
| ClassII/Maverick | 64,882 | 16,729,014 | 1.58 |
| ClassII/TIR | 444,836 | 110,668,046 | 10.46 |

**Table S6:** Summary of the gene prediction results

| Method | Software | Species | Gene number |
| --- | --- | --- | --- |
| Ab initio | Genscan | - | 30,011 |
|  | Augustus | - | 37,757 |
|  | GlimmerHMM | - | 190,287 |
|  | GeneID | - | 36,389 |
|  | SNAP | - | 72,674 |
| Homology-based | GeMoMa | *Danio rerio* | 16,546 |
|  |  | *Crassostrea gigas* | 29,110 |
|  |  | *Crassostrea virginica* | 28,275 |
|  |  | *Mizuhopecten yessoensis* | 28,700 |
| Transcriptome-based | PASA | - | 80,463 |
|  | GeneMarkS-T | - | 53,118 |
|  | TransDecoder | - | 20,571 |
| Integration | EVM | - | 38,841 |

**Table S7:** Statistics of gene annotation to different databases

| Annotation database | Annotated number | Percentage (%) | 100≤Protein length<300 | Protein length≥300 |
| --- | --- | --- | --- | --- |
| GO_Annotation | 7,489 | 19.28 | 2,243 | 5,119 |
| KEGG_Annotation | 12,757 | 32.84 | 3,466 | 9,144 |
| KOG_Annotation | 18,233 | 46.94 | 4,642 | 13,426 |
| TrEMBL_Annotation | 32,280 | 83.11 | 10,097 | 21,841 |
| Nr_Annotation | 32,382 | 83.37 | 10,170 | 21,858 |
| All_Annotated | 32,591 | 83.91 | 10,275 | 21,957 |

**Table S12:** Summary of gene features in five bivalves

| Species | Multi ortholog | Other ortholog | Single ortholog | Special |
| --- | --- | --- | --- | --- |
| *Bathymodiolus platifrons* | 4,554 | 4,307 | 146 | 23,021 |
| *Corbicula fluminea* | 5,754 | 4,692 | 146 | 25,878 |
| *Crassostrea gigas* | 8,728 | 30,181 | 146 | 18,847 |
| *Crassostrea virginica* | 11,433 | 34,131 | 146 | 13,685 |
| *Ruditapes philippinarum* | 4,545 | 2,446 | 146 | 14,480 |
