# Supplementary figures and images for: Dissecting the chromosome-level genome of the Asian Clam (Corbicula fluminea)
Source: Sci Rep. 2021 Jul 22;11:15021. doi: 10.1038/s41598-021-94545-2 (PMC8298618; doi:10.1038/s41598-021-94545-2)

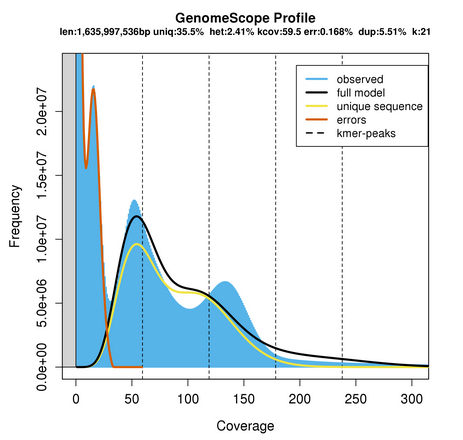

Supplement: Supplementary file 2 — Supplementary Figure S1. [file 41598_2021_94545_MOESM2_ESM.png]

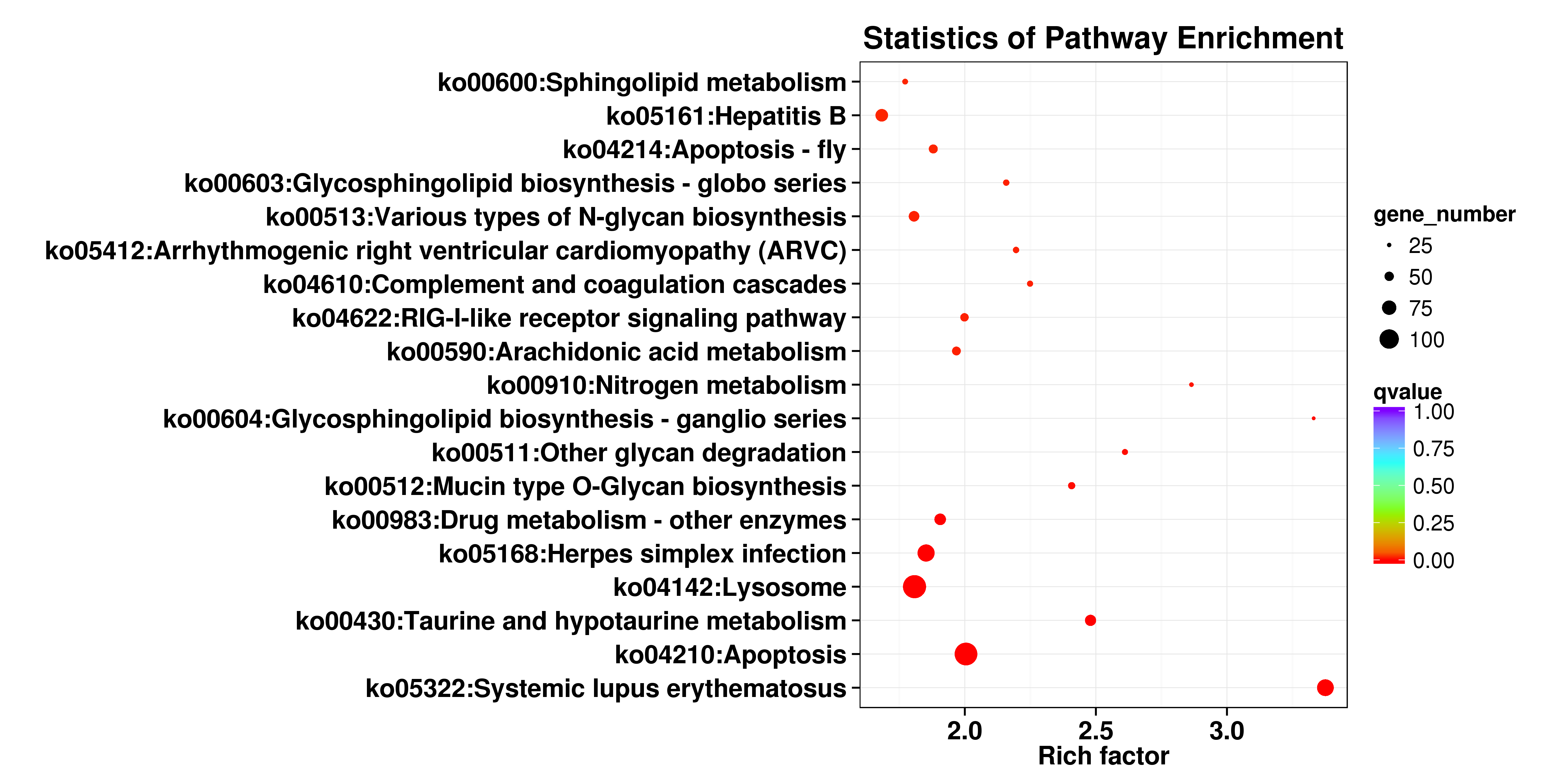

Supplement: Supplementary file 3 — Supplementary Figure S2. [file 41598_2021_94545_MOESM3_ESM.png]
